# Study on the Correlation Between CT Features and Vascular Tumor Thrombus Together With Nerve Invasion in Surgically Resected Lung Adenocarcinoma

**DOI:** 10.3389/fsurg.2022.931568

**Published:** 2022-06-28

**Authors:** Yu Song, Daiwen Chen, Duohuang Lian, Shangwen Xu, Hui Xiao

**Affiliations:** ^1^ Department of Diagnostic Radiology, 900 Hospital of the Joint Logistics Team, Fuzhou, China; ^2^ Department of Cardiothoracic Surgery, 900 Hospital of the Joint Logistics Team, Fuzhou, China

**Keywords:** lung adenocarcinoma, vascular carcinoma thrombus, nerve invasion, surgical excision, CT features

## Abstract

**Background:**

We aimed to analyze the relationship between pulmonary adenocarcinoma patients with vascular tumor thrombus and nerve invasion and different CT features.

**Methods:**

The preoperative CT scanning data of 86 patients with lung adenocarcinoma who underwent surgical resection in our hospital from January 2020 to January 2022 were analyzed in the form of retrospective analysis. The CT images of all patients were observed, and the relationship between them and vascular tumor thrombus and nerve invasion of lung adenocarcinoma was analyzed. At the same time, the sensitivity, specificity, and accuracy of enhanced CT and plain CT were compared to evaluate the diagnostic efficacy of both.

**Results:**

The results showed that the vascular tumor thrombus of lung adenocarcinoma was mainly related to the solid components and lobulated and calcified tumors in CT images, and the nerve invasion of lung adenocarcinoma was mainly related to the tumors with bronchial inflation sign in CT images (*P* < 0.05). The sensitivity, specificity, and accuracy of enhanced CT in the diagnosis of vascular tumor thrombus were 78.26%, 96.83%, and 91.86%, respectively, and the sensitivity, specificity, and accuracy in the diagnosis of nerve invasion were 75.00%, 98.72%, and 96.51%, respectively. The sensitivity, specificity, and accuracy of plain CT in the diagnosis of vascular tumor thrombus were 43.48%, 92.06%, and 79.07%, respectively, and the sensitivity, specificity, and accuracy in the diagnosis of nerve invasion were 25.00%, 94.87%, and 88.37%, respectively. The contrast showed that the sensitivity and accuracy of enhanced CT were higher than those of plain CT (*P* < 0.05), but the difference of specificity was not obvious (*P* > 0.05).

**Conclusions:**

Solid components and lobulated and calcified tumors in CT signs are closely related to vascular tumor thrombus of lung adenocarcinoma, while patients with bronchial inflation sign are related to nerve invasion.

## Introduction

Lung cancer is a common malignant tumor in clinical practice. Its incidence rate and mortality rate rank first in China. According to the relevant survey, the incidence rate of lung cancer is increasing year by year in China. The incidence rate in male population is the highest, and lung adenocarcinoma is the most common, accounting for 45.5% (1) of all lung cancer patients. Surgical resection is an effective way to treat lung adenocarcinoma, but the survival and recurrence of patients are still difficult to guarantee. It has been reported that the survival time and disease recurrence of patients with lung adenocarcinoma after the operation are mainly affected by vascular tumor thrombus, and vascular tumor thrombus and nerve invasion are also important risk factors for lymph node metastasis of lung adenocarcinoma (2). At present, the clinical diagnosis of vascular cancer thrombus and nerve invasion is mostly confirmed by postoperative pathology, but it is the lack of predictability that is easy to cause patients to miss the best operation opportunity. Therefore, it is very important to find a more effective diagnosis method. Chest CT is a routine method for clinical screening of lung lesions. In recent years, with the continuous improvement and optimization of CT technology, the information presented by this image is much rich and accurate. Therefore, it is possible to predict vascular tumor thrombus and nerve invasion of lung adenocarcinoma by preoperative enhanced CT (3). This paper mainly studies the correlation between different CT features and vascular tumor thrombus and nerve invasion of lung adenocarcinoma to clarify the value of CT scanning in the prognosis of patients.

## Patients and Methods

### Patients

A total of 86 patients with lung adenocarcinoma who underwent surgical resection in our hospital from January 2020 to January 2022 were selected, and their clinical data were analyzed retrospectively. All patients underwent CT scanning before operation and were confirmed by pathological results after operation. There were 45 males and 31 females; the lower limit of age was 35 years, the upper limit was 85 years, and the average age was 55.38 ± 8.72 years; the lesions were located in the right lung in 44 cases and in the left lung in 44 cases; and there were 71 cases of invasive type and 15 cases of microinvasive type. This study was approved by the ethics committee of the 900 Hospital of the Joint Logistics Team. Signed written informed consent was obtained from all participants before the study.

### Inclusion and Exclusion Criteria

Inclusion criteria (4) are as follows: (1) lung adenocarcinoma confirmed by postoperative histopathological examination; (2) the chest was examined by enhanced CT before operation, and there were complete image data; (3) patients did not receive any antitumor drugs before operation; and (4) there were no other malignant tumors. Exclusion criteria are as follows: (1) patients with contrast medium allergy and unable to complete CT scan; (2) combined with other malignant tumors and treated with antitumor therapy; (3) the quality of enhanced CT image is too poor for accurate analysis; and (4) the CT image was more than 1 month away from the operation time.

## Methods

The chest CT scanning equipment was Ge VCT 64 row, Lianying uct780 64 row, Ge Discovery HD 750, and ingenuity flex16 row. The tube voltage of the scanning instrument was set to 120 kv, the rotating speed was 0.7 s per revolution, the matrix was  512 × 512, and the layer thickness was set to 5 mm. During the scan, the patient laid on his back on the bed and was asked to hold his breath and lift his hands above his head. After completing the plain CT scan, the enhanced scan was started with a delay of 30s. During the enhanced scan, the patient was injected with 80 ml of contrast agent iohexol (350 mg/ml) through a high-pressure syringe. The injection site was in the vein of the elbow, and 3 ml was injected every second. After scanning, the CT images of all patients were reconstructed into images with a layer thickness of 1.25 mm and uploaded to the imaging workstation to analyze the lesions.

### Image Analysis

CT images were discussed and analyzed by two experienced radiologists, mainly analyzing multiple CT signs such as boundary, composition, lobulation sign, bronchial inflation sign, pleural thickening, calcification, pleural depression sign, vascular bundle sign, vacuole, peripheral emphysema, and pleural effusion.

### Statistical Analysis

Statistical Product and Service Solutions (SPSS) 22.0 (IBM, Armonk, NY, USA) was applied for statistical analysis. Each categorical variable was expressed as enumeration data [*n*(%)] and passed the *χ*^2^ value test. When the results were compared, *P *< 0.05 indicated a significant difference.

## Results

### Postoperative Pathology and CT Diagnosis Results

After histopathological examination of 86 patients with lung adenocarcinoma, 23 (26.74%) were positive for vascular tumor thrombus and 63 (73.26%) were negative and 8 (9.30%) were positive for nerve invasion and 78 (90.70%) were negative. Enhanced CT scan showed that 20 cases (23.26%) were positive for vascular tumor thrombus and 66 cases (76.74%) were negative and 7 cases (8.14%) were positive for nerve invasion and 79 cases (91.86%) were negative. CT scan showed that 15 cases (17.44%) were positive for vascular tumor thrombus and 71 (82.56%) were negative and 6 (6.98%) were positive for nerve invasion and 80 (93.02%) were negative.

### Relationship Between Different CT Features of Lung Adenocarcinoma and Vascular Tumor Thrombus

Lung adenocarcinoma patients with solid components and lobulated and calcified tumors were all significantly correlated with vascular tumor thrombus (*P *< 0.05), while other signs had no significant correlation (*P *> 0.05) (Table 1).

**Table 1:** Relationship between different CT features of lung adenocarcinoma and vascular tumor thrombus [*n* (%)].

CT signs	Positive (*N* = 23)	Negative (*N* = 63)	*χ* ^2^	*P*
Clear boundaries	14(60.87)	47(74.60)	1.541	0.214
Solid ingredient	20(86.96)	40(63.49)	4.398	0.036
Lobulated sign	19(82.61)	37(58.73)	4.449	0.040
Glitches	17(73.91)	43(68.25)	0.256	0.613
Vascular bundle sign	20(96.96)	49(77.78)	0.895	0.344
Air bronchus sign	16(69.57)	42(66.67)	0.064	0.800
Vacuoles	10(43.48)	23(36.51)	0.346	0.556
Adjacent pleura thickening	18(78.26)	45(71.43)	0.401	0.526
Pleural indentation sign	21(91.30)	46(73.02)	3.274	0.070
With calcification	6(26.09)	5(7.94)	4.976	0.026
Pleural effusion	0(0.00)	4(6.35)	1.532	0.216
Peripheral emphysema	5(21.74)	7(11.11)	1,585	0.208
Multiple enlarged lymph nodes	4(17.39)	6(9.52)	1.015	0.314
Calcified lymph nodes	5(21.74)	9(14.29)	0.687	0.407

### Relationship Between Different CT Signs and Nerve Invasion in Patients With Lung Adenocarcinoma

After analysis, it was found that the nerve invasion of surgically resected lung adenocarcinoma was mainly related to tumors with air bronchi signs (*P *< 0.05), and there was no significant difference with other signs and no correlation (*P *> 0.05) (Table 2).

**Table 2:** Relationship between different CT signs and nerve invasion in patients with lung adenocarcinoma [*n* (%)].

CT signs	Positive (*N* = 8)	Negative (*N* = 78)	χ^2^	*P*
Clear boundaries	6(75.00)	70(89.74)	1.535	0.215
Solid ingredient	8(100.00)	68(87.18)	1.161	0.281
Lobulated sign	7(87.50)	54(69.23)	1.175	0.278
Glitches	8(100.00)	71(91.03)	0.782	0.377
Vascular bundle sign	6(75.00)	67(85.90)	0.672	0.413
Air bronchus sign	8(100.00)	0(66.67)	86.000	<0.001
Vacuoles	3(37.50)	24(30.77)	0.153	0.696
Adjacent pleura thickening	8(100.00)	65(83.33)	1.571	0.210
Pleural indentation sign	7(87.50)	66(84.62)	0.047	0.828
With calcification	2(25.00)	8(10.26)	1.535	0.215
Pleural effusion	0(0.00)	9(11.54)	1.031	0.310
Peripheral emphysema	1(12.50)	5(6.41)	0.415	0.520
Multiple enlarged lymph nodes	0(0.00)	3(3.85)	0.319	0.572
Calcified lymph nodes	4(50.00)	20(25.64)	2.140	0.144

### Comparison of Contrast-Enhanced CT and Plain CT in the Diagnosis of Vascular Tumor Thrombus in Lung Adenocarcinoma

Compared with plain CT, enhanced CT scanning sensitivity and accuracy were significantly higher (*P* < 0.05), and there was no difference in specificity between the two scanning methods (*P* > 0.05) (Tables 3 and 4).

**Table 3:** Comparison of contrast-enhanced CT and plain CT in the diagnosis of vascular tumor thrombus in lung adenocarcinoma [*n* (%)].

Item		Postoperative pathology		Total
		Positive	Negative	
Enhanced CT	Positive	18	2	20
	Negative	5	61	66
Total		23	63	86
Plain CT	Positive	10	5	15
	Negative	13	58	71
Total		23	63	86

**Table 4:** Comparison of contrast-enhanced CT and plain CT in the diagnosis of vascular tumor thrombus in lung adenocarcinoma [*n* (%)].

Item	Sensitivity	Specificity	Accuracy
Enhanced CT	78.26%(18/23)	96.83%(61/63)	91.86%(79/86)
Plain CT	43.48%(10/23)	92.06%(58/63)	79.07%(68/86)
χ^2^	5.841	1.361	5.663
*P*	0.016	0.243	0.017

### Comparison of Contrast-Enhanced CT and Plain CT in the Diagnosis of Nerve Invasion in Lung Adenocarcinoma

Compared with plain CT, the sensitivity and accuracy of enhanced CT were significantly higher (*P* < 0.05), but there was no significant difference in specificity between the two (*P* > 0.05) (Tables 5 and 6).

**Table 5:** Comparison of the results of contrast-enhanced and non-enhanced CT in the diagnosis of nerve invasion in lung adenocarcinoma [*n* (%)].

Item		Postoperative pathology		Total
		Positive	Negative	
Enhanced CT	Positive	6	1	7
	Negative	2	77	79
Total		8	78	86
Plain CT	Positive	2	4	6
	Negative	6	74	80
Total		8	78	86

**Table 6:** Comparison of the results of contrast-enhanced and non-enhanced CT in the diagnosis of nerve invasion in lung adenocarcinoma [*n* (%)].

Item	Sensitivity	Specificity	Accuracy
Enhanced CT	75.00%(6/8)	98.72%(77/78)	96.51%(83/86)
Plain CT	25.00%(2/8)	94.87%(74/78)	88.37%(76/86)
χ^2^	4.000	1.860	4.077
*P*	0.045	0.173	0.043

## Discussion

Lung adenocarcinoma is highly malignant and can pose a serious threat to the life safety of patients. Although the clinical resection can temporarily save the lives of patients, the problem of postoperative survival and recurrence still puzzles the medical community. Studies have found that vascular tumor thrombus and nerve invasion are independent risk factors leading to lymph node metastasis of lung adenocarcinoma and affecting patients’ recurrence (5, 6). Vascular tumor thrombus refers to the long-term effect of various reasons, which makes tumor cells invade blood vessels or lymphatic vessels and finally leads to the formation of tumor thrombus. Vascular tumor thrombus is a typical sign of tumor cells invading the vascular system and a pioneer condition for inducing lymph node metastasis (7, 8). Therefore, it is necessary to strengthen preoperative diagnosis, which is of great significance to reduce postoperative recurrence and prolong the survival of patients.

CT is currently a commonly used imaging method for clinical diagnosis of various diseases, with high sensitivity and specificity. Using it in the preoperative examination of lung adenocarcinoma can determine the deterioration by analyzing the changes of various signs (9–11). However, the research on the relationship between CT features and vascular tumor thrombus and nerve invasion of lung adenocarcinoma is still rare in the medical community. Therefore, based on the above shortcomings, this paper uses CT scanning for patients with pulmonary adenocarcinoma before operation. The results show that patients with vascular tumor thrombus are mainly closely related to CT features such as solid nodule, lobulation, and calcification, while there is a certain correlation between nerve invasion of pulmonary adenocarcinoma and patients with bronchial inflation sign (*P* < 0.05). This result is basically consistent with a previous study (12). In its report, the analysis of preoperative chest CT images of patients with surgically resected lung adenocarcinoma found that patients with vascular tumor thrombus were mostly accompanied by solid nodules, lobulated nodules, and calcified nodules, while patients with nerve invasion were mostly biased toward bronchial inflation sign tumors. This shows that CT signs were indeed related to vascular tumor thrombus and nerve invasion of lung adenocarcinoma. Preoperative enhanced CT scanning can effectively predict the condition and prognosis of patients (13, 14).

Usually, the malignant degree of solid nodules is higher than that of pure ground glass nodules and mixed ground glass nodules, and the proportion of solid components will increase significantly with the deepening of tumor infiltration. Therefore, patients with lung adenocarcinoma with solid nodules are more likely to have vascular tumor thrombus and have a high postoperative recurrence rate and poor prognosis (15). Lobulated lung adenocarcinoma is more common in solid nodules, which may be related to the great difference of tumor cell differentiation. Malignant tumors are very prone to calcification, but even after calcification, they are still likely to continue to grow, spread, and metastasize. Therefore, this sign has a certain value in predicting the prognosis of patients (16, 17). However, due to the small number of positive cases of calcification in this paper, it has little reference significance. Accompanied by bronchial inflation sign, it is usually considered as alveolar inflammatory exudation, but it does not cause significant damage to the bronchus. In the background of consolidation, the bronchial sign with air usually appears in independent nodules. This sign should be highly vigilant against malignant tumors (18).

To further understand the predictive value of different ways of CT scanning (enhanced CT and plain CT) for vascular tumor thrombus and nerve invasion of lung adenocarcinoma, the diagnostic efficacy of the two was compared. The results showed that the diagnostic sensitivity and accuracy of enhanced CT were significantly higher than those of plain CT (*P* < 0.05), but there was no significant difference in the diagnostic specificity between the two. The results suggested that enhanced CT is more valuable in the diagnosis of vascular tumor thrombus and nerve invasion of lung adenocarcinoma than plain CT, which can guide the clinical development of effective diagnosis and treatment plan. The application of spiral CT effectively improves the diagnostic accuracy, and the reconstructed CT image can observe the lesion characteristics from multiple angles and objectively analyze the lesion characteristics to improve the diagnostic accuracy and guide the clinical rational treatment of diseases (7, 19).

It is worth noting that there are still some deficiencies in this paper, such as small sample size and little reference significance of some conclusions. The pathological type was single, and no clear pathological distinction was made; The clinical stage of lung adenocarcinoma was not clear, so it still needed to be strengthened and improved in future research.

## Conclusion

The tumors with solid components and lobulation and calcification in CT features are closely related to the vascular tumor thrombus of lung adenocarcinoma, and the tumors with bronchial inflation sign are related to the nerve invasion of lung adenocarcinoma. Therefore, it is necessary to strengthen the prediction and diagnosis of patients with lung adenocarcinoma before operation.

## Data Availability

The original contributions presented in the study are included in the article/Supplementary Material, further inquiries can be directed to the corresponding author/s.

## References

[1] Li X Zhang W Yu Y Zhang G Zhou L Wu Z CT features and quantitative analysis of subsolid nodule lung adenocarcinoma for pathological classification prediction. BMC Cancer. (2020) 20(1):60. 10.1186/s12885-020-6556-6 31992239 PMC6986053

[2] Sandler AB Johnson DH Herbst RS. Anti-vascular endothelial growth factor monoclonals in non-small cell lung cancer. Clin Cancer Res. (2004) 10(12 Pt 2):4258s–62s. 10.1158/1078-0432.CCR-040023 15217970

[3] Wu T Zhou F Soodeen-Lalloo AK Yang X Shen Y Ding X The association between imaging features of TSCT and the expression of PD-L1 in patients with surgical resection of lung adenocarcinoma. Clin Lung Cancer. (2019) 20(2):e195–207. 10.1016/j.cllc.2018.10.012 30514666

[4] Li Z Li F Pan C He Z Pan X Zhu Q Tumor cell proliferation (Ki-67) expression and its prognostic significance in histological subtypes of lung adenocarcinoma. Lung Cancer. (2021) 154:69–75. 10.1016/j.lungcan.2021.02.009 33626488

[5] Wang YN Yao S Wang CL Li MS Sun LN Yan QN Clinical significance of 4l lymph node dissection in left lung cancer. J Clin Oncol. (2018) 36(29):2935–42. 10.1200/JCO.2018.78.7101 30148659

[6] Rena O Papalia E Ruffini E Casadio C Filosso PL Oliaro A Stage I pure bronchioloalveolar carcinoma: recurrences, survival and comparison with adenocarcinoma of the lung. Eur J Cardiothorac Surg. (2003) 23(3):409–14. 10.1016/s1010-7940(02)00830-8 12614815

[7] Wang Y Jing L Wang G. Risk factors for lymph node metastasis and surgical methods in patients with early-stage peripheral lung adenocarcinoma presenting as ground glass opacity. J Cardiothorac Surg. (2020) 15(1):121. 10.1186/s13019-020-01167-2 32782020 PMC7422532

[8] Zhu Y Hou D Lan M Sun X Ma X. A comparison of ultra-high-resolution CT target scan versus conventional CT target reconstruction in the evaluation of ground-glass-nodule-like lung adenocarcinoma. Quant Imaging Med Surg. (2019) 9(6):1087–94. 10.21037/qims.2019.06.09 31367562 PMC6629564

[9] Yan J Wang H Zhou H He H Qiu L Wang Z. Correlation between expression of Ki-67 and MSCT signs in different types of lung adenocarcinoma. Medicine (Baltimore). (2020) 99(2):e18678. 10.1097/MD.0000000000018678 31914061 PMC6959960

[10] Eriguchi D Shimada Y Imai K Furumoto H Okano T Masuno R Predictive accuracy of lepidic growth subtypes in early-stage adenocarcinoma of the lung by quantitative CT histogram and FDG-PET. Lung Cancer. (2018) 125:14–21. 10.1016/j.lungcan.2018.08.027 30429012

[11] Jeong CJ Lee HY Han J Jeong JY Lee KS Choi YL Role of imaging biomarkers in predicting anaplastic lymphoma kinase-positive lung adenocarcinoma. Clin Nucl Med. (2015) 40(1):e34–9. 10.1097/RLU.0000000000000581 25243942

[12] Xu Y Ji W Hou L Lin S Shi Y Zhou C Enhanced CT-based radiomics to predict micropapillary pattern within lung invasive adenocarcinoma. Front Oncol. (2021) 11:704994. 10.3389/fonc.2021.704994 34513686 PMC8429899

[13] Yang X Wang G Gu R Xu X Zhu G. A signature of tumor DNA repair genes associated with the prognosis of surgically-resected lung adenocarcinoma. Peerj. (2020) 8:e10418. 10.7717/peerj.10418 33304656 PMC7700735

[14] Wang T Yang Y Liu X Deng J Wu J Hou L Primary invasive mucinous adenocarcinoma of the lung: prognostic value of CT imaging features combined with clinical factors. Korean J Radiol. (2021) 22(4):652–62. 10.3348/kjr.2020.0454 33236544 PMC8005341

[15] Grant-Freemantle MC Bass GA Butt WT Gillis AE. Splenectomy for isolated splenic metastasis from primary lung adenocarcinoma. BMJ Case Rep. (2020) 13(3):e233256. 10.1136/bcr-2019-233256 32152067 PMC7064137

[16] Wang Q Ba W Yin K Shen J Jiang G Liang Y Predicting lung adenocarcinoma invasiveness by measurement of pure ground-glass nodule roundness by using multiplanar reformation: a retrospective analysis. Clin Radiol. (2022) 77(1):e20–6. 10.1016/j.crad.2021.10.007 34772486

[17] Tao S Yu J Xu Y Deng B Sun T Hu P PC4 induces lymphangiogenesis dependent VEGF-C/VEGF-D/VEGFR-3 axis activation in lung adenocarcinoma. Am J Cancer Res. (2015) 5(6):1878–89. PMID: ; PMCID: 26269750 PMC4529610

[18] Sun Y Li C Jin L Gao P Zhao W Ma W Radiomics for lung adenocarcinoma manifesting as pure ground-glass nodules: invasive prediction. Eur Radiol. (2020) 30(7):3650–9. 10.1007/s00330-020-06776-y 32162003 PMC7305264

[19] Hu F Huang H Jiang Y Feng M Wang H Tang M Discriminating invasive adenocarcinoma among lung pure ground-glass nodules: a multi-parameter prediction model. J Thorac Dis. (2021) 13(9):5383–94. 10.21037/jtd-21-786 34659805 PMC8482342

